# A concept for single-shot volumetric fluorescence imaging via orthogonally polarized excitation lattices

**DOI:** 10.1038/s41598-019-42743-4

**Published:** 2019-04-23

**Authors:** Florian Ströhl, Clemens F. Kaminski

**Affiliations:** 1 Department of Chemical Engineering and Biotechnology, University of Cambridge, CB3 0AS Cambridge, UK; 2 Present Address: Department of Physics and Technology, UiT-The Arctic University of Norway, 9037 Tromsø, Norway

**Keywords:** Wide-field fluorescence microscopy, Fluorescence imaging, Biomedical engineering

## Abstract

The deconvolution of widefield fluorescence images provides only guesses of spatial frequency information along the optical axis due to the so called missing cone in the optical transfer function. Retaining the single-shot imaging speed of deconvolution microscopy while gaining access to missing cone information is thus highly desirable for microscopy of volumetric samples. Here, we present a concept that superimposes two orthogonally polarized excitation lattices with a phase-shift of p between them. In conjunction with a non-iterative image reconstruction algorithm this permits the restoration of missing cone information. We show how fluorescence anisotropy could be used as a method to encode and decode the patterns simultaneously and develop a rigorous theoretical framework for the method. Through *in-silico* experiments and imaging of fixed biological cells on a structured illumination microscope that emulates the proposed setup we validate the feasibility of the method.

## Introduction

Optical sectioning (OS) is a key requirement to provide contrast in volumetric imaging and is commonly realized via confocal detection or two-photon excitation. These scanning microscopies are significantly slower than widefield alternatives, such as spinning disk, light-sheet, or structured illumination microscopy, SIM^1^. The latter technique offers computational sectioning and its earliest form, known as optical sectioning SIM, OS-SIM, introduced by Neil *et al*.^2^, removes out-of-focus light via a homodyne detection scheme implemented as

1 $${i}_{OS}=\sqrt{{({i}_{1}-{i}_{2})}^{2}+{({i}_{1}-{i}_{3})}^{2}+{({i}_{2}-{i}_{3})}^{2}}.$$

Here, *i*_1_ to *i*_3_ represent images taken under sinusoidal excitation patterns with different phases but same spatial frequency and orientation. Although appealing due to its simplicity, this OS technique does not realize a linear reconstruction procedure, i.e. the geometric sum of difference images yields a final image *i*_*OS*_ that is not shift-invariant. Hence, it is not possible to define a point spread function, PSF^3^ for the sectioned image. Super-resolution (SR) SIM can also provide OS, which was outlined by Gustafsson *et al*. for 3-beam SIM (also called 3D SIM)^4^ and Wicker for 2-beam SIM^5^ (also called 2D SIM). Despite the linear processing procedures of SR SIM, the comparatively many raw frames (9–15) render these approaches less useful when minimal excitation doses or maximal frame-rates are desired. To address this problem, Wicker and Heintzmann pioneered the idea of single shot optical sectioning (ssOS), which builds on the homodyne OS SIM concept^3^. Using the orthogonal system of horizontally, vertically, and un-polarized light in conjunction with structured illumination, they showed that it is possible in theory to acquire three independent raw frames within a single acquisition and to process them into an optically sectioned image. Assuming no rotational diffusion and reorientation of fluorescent dipoles during image acquisition as well as sufficient modulation contrast of the excitation pattern, the three raw frames can be separated. In practice the achievable modulation contrast of this approach is expected to be low as the necessary system of orthogonal polarization directions is based on a small angle approximation (Müller matrix formalism) and no experimental demonstration was shown. Optical sectioning can also be obtained from two images, one widefield and one structured illumination or^6^ speckle illumination^7^ image, which are combined computationally. As the involved algorithms require high-pass and low-pass filtering, the latter two approaches are commonly referred to as HiLo microscopy. In contrast to OS-SIM, HiLo microscopy appears well-suited for single-shot sectioning as the intuitive orthogonal system of vertical and horizontal polarizations offers a stronger modulation contrast than the triplet of horizontally, vertically, and un-polarized light. However, HiLo also requires non-linear processing steps and the sample is illuminated non-homogeneously. Here, we present the framework for an OS method, which relies on purely linear processing steps from two structured illumination images. The proposed method can be realized with a uniform illumination field across the sample. Using polarization coded patterns, the method could be implemented into an imaging technique, that captures multiple sectioned planes across a sample volume within a single shot acquisition.

## Theoretical Framework

Three aspects of the proposed ssOS method shall be elucidated in greater detail. First, a non-iterative reconstruction algorithm is derived for data sets obtained with striped illumination patterns from coherent excitation beams. We then present feasibility calculations on obtainable modulation contrasts via polarization encoded illumination patterns. Finally, a conceptual optical set-up is outlined, which focuses on the generation of orthogonally polarized excitation lattices and volumetric image capture.

### Reconstruction algorithm

**Figure 1:**
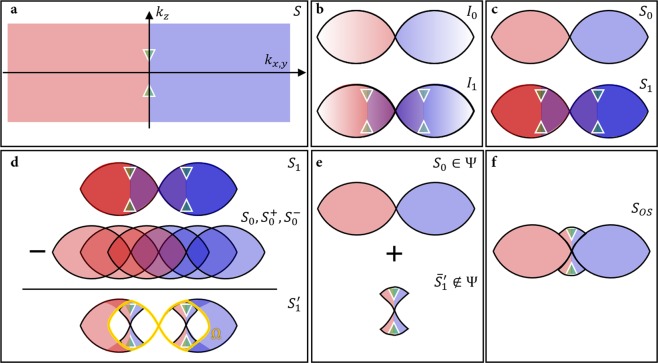
Illustration of the method. (**a**) Example spectrum of an object with distinct features (green triangles) along the optical axis, symbolizing the missing cone. (**b**) Spectra transmitted by widefield imaging (top) and structured illumination (SI) imaging (bottom). (**c**) Corresponding spectra after deconvolution (denoted as *S*_0_ and *S*_1_, respectively). (**d**) The missing cone components can be retrieved through subtraction of several shifted widefield spectra *S*_0_ from the SI spectrum *S*_1_. Superscripts denote shift directions with respect to original spectrum *S*_0_. (**e**) Combination of the original widefield spectrum with the averaged cone component yields (**f**) the sectioned spectrum *S*_*OS*_.

Equations 2–8 constitute the theoretical framework of ssOS. Their meaning is illustrated in Fig. 1. Let us assume that two raw images, *i*′ and *i*″, are taken with sinusoidal excitation patterns at spatial frequency *p*. Both feature equal modulation contrast *n* and there is a phase shift of between them, i.e. the sum of both exposures *s* is spatially constant. *ϕ*_0_ denotes an arbitrary overall phase offset for pattern *i*′. We note that taking the average of both images yields a widefield image *i*_0_, whilst any of the individual raw images can be used directly as a structured illumination image *i*_1_. Let us denote the point spread function by *h*, and all variables in Fourier space via capitalization of their object space analogues. In Fourier space, the spectra *I*_0_ and *I*_1_ of the two images *i*_0_ and *i*_1_ then read

2 $${I}_{0}(k)=\frac{I^{\prime} (k)+I^{\prime\prime} (k)}{2}=H(k)\times S(k)$$

3 $${I}_{1}(k)=I^{\prime} (k)=H(k)\times (S(k)+n{e}^{-i{\varphi }_{0}}S(k-p)+n{e}^{i{\varphi }_{0}}S(k+p)).$$

It can be seen that the spatial frequencies of the sample are truncated by the limited support of the OTF, *H*. Furthermore, the low frequency part of the OTF contains mostly out-of-focus light, which is unwanted signal. This is clear when the 2D OTF is viewed as the *k*_*z*_ projection of the 3D OTF, which suffers from the so-called missing cone of spatial frequencies along the *k*_*z*_ axis. To undo the weighting of the OTF within its support, one can use Wiener filtering or Richardson-Lucy deconvolution of *I*_0_ with *H*, depending on the dominant noise model. In the ideal, noise-free case, we thus obtain a high-fidelity truncated version of the sample spectrum *S*, albeit with out-of-focus corrupted values at low spatial frequencies. Let us define the 3D support of *H* as Ψ. Then the spectrum *S*_0_ is

4 $${S}_{0}(k)=(\begin{array}{cc}S(k) & {\rm{k}}\in {\rm{\Psi }}\\ 0 & {\rm{otherwise}}{\rm{.}}\end{array}$$

Similarly, the structured illumination spectrum *S*_1_ can be obtained from image *I*_1_ as the truncated and high-pass filtered sample spectrum

5 $${S}_{1}(k)=(\begin{array}{ll}S(k)+n{e}^{-i{\varphi }_{0}}S(k-p)+n{e}^{+i{\varphi }_{0}}S(k+p) & {\rm{k}}\in {\rm{\Psi }}\\ 0 & {\rm{otherwise}}{\rm{.}}\end{array}$$

As can be seen in Fig. 1c, the filtered spectrum *S*_1_ contains the frequency information from the missing cone, provided by the shifted copies of the original sample spectrum *S*. The remaining challenge lies in the un-mixing of these spatial frequency components from the superposition of the shifted spectra in *S*_1_. Subtracting three shifted copies of *S*_0_, positioned at spatial frequencies [−*p*, 0, +*p*] and with correct weighting, from *S*_1_, yields an intermediate spectrum ${S}_{1}^{^{\prime} }$ that contains the missing cone components positioned at −*p* and +*p*. The required weighting is determined by the phase and modulation contrast of the illumination structure (which can be retrieved from the raw data^5,8,9^):

6 $${S}_{1}^{^{\prime} }(k)={S}_{1}(k)-{S}_{0}(k)-n{e}^{-i{\varphi }_{0}}{S}_{0}(k-p)-n{e}^{i{\varphi }_{0}}{S}_{0}(k+p\mathrm{).}$$

Shifting and weighted averaging of the missing cone components then yield the spatial frequencies along *k*_*z*_. Note that residual high spatial frequency fragments that were translated laterally beyond the cut-off are removed from ${S}_{1}^{^{\prime} }$ before shifting and weighting by restricting ${S}_{1}^{^{\prime} }$ to a region Ω defined as the overlap of Ψ(*k* − *p*) and Ψ(*k* + *p*). With Ω being unity across its support and null elsewhere, the missing cone component ${\bar{S}}_{1}^{^{\prime} }$ is obtained via

7 $$\begin{array}{l}\begin{array}{rcl}{\bar{S}}_{1}^{^{\prime} }(k) & = & \frac{1}{2}\frac{1}{n{e}^{-i{\varphi }_{0}}}[{S}_{1^{\prime} }(k)\times {\rm{\Omega }}(k)]\otimes \delta (k-p)\\  &  & +\,\frac{1}{2}\frac{1}{n{e}^{+i{\varphi }_{0}}}[{S}_{1^{\prime} }(k)\times {\rm{\Omega }}(k)]\otimes \delta (k+p\mathrm{).}\end{array}\end{array}$$

Note that due to the shape of Ψ, the cone components of ${S}_{1}^{^{\prime} }(k-p)$ and ${S}_{1}^{^{\prime} }(k+p)$ do not overlap perfectly. In regions where only one of the two components is non-zero, no averaging is performed. The full, optically sectioned, spectrum can be reconstructed by combining the missing cone components of ${\bar{S}}_{1}^{^{\prime} }$ and the widefield spectrum *S*_0_:

8 $${S}_{OS}={S}_{0}{|}_{\in {\rm{\Psi }}}+{\bar{S}}_{1}^{^{\prime} }{|}_{\notin {\rm{\Psi }}}.$$

**Figure 2:**
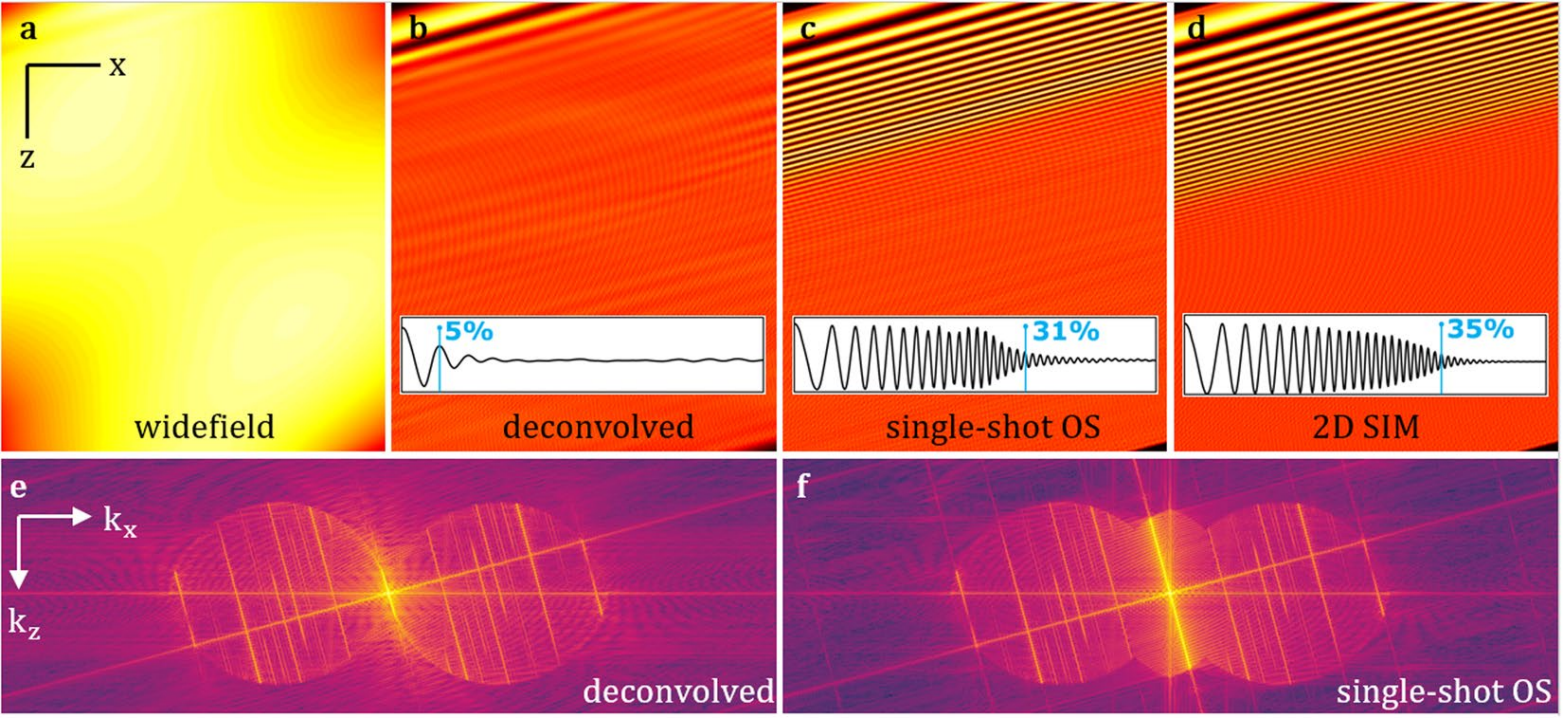
Volumetric imaging was simulated on a sample featuring stripes at a 15° tilt with respect to the optical axis. The spatial frequency of the sample pattern increases from zero at the top to the axial cut-off frequency for confocal imaging at the bottom. (**a**) Widefield microscopy is not able to resolve any features but, (**b**) a small portion of spatial frequencies become visible after deconvolution. (**c**) A much greater extent of axial spatial frequency information can be recovered with single-shot OS and, (**d**) with conventional 2D SIM implementing optical sectioning. Inlays show line profiles with marks where the modulation drops below 25%. Percentages indicate spatial frequencies retained with respect to confocal microscopy. (**e**) The cone of spatial frequencies along the axial frequency direction is missing in the deconvolved widefield spectrum but, (**f**) successfully reconstructed in single-shot OS. Scale in (**a**–**d**) is 10 m. Imaging parameters provided as SI.

The optically sectioned image *s*_*OS*_ is then obtained as the inverse Fourier transform of *S*_*OS*_. To validate the proposed sectioning approach, a sample was simulated that features a spatial frequency chirp along an axis tilted at 15° with respect to the *z* direction (full simulation parameters are given in materials and methods). The sample was then imaged *in silico* in widefield mode, followed by deconvolution with a Wiener filter. Using a simulated structured illumination pattern, the proposed ssOS algorithm was employed. For comparison, standard 2-beam SIM with an implementation of optical sectioning^5^ was also performed. Resulting images in *x*-*z* are shown in Fig. 2a–d. The sectioning capability is clearly visible in the object space images. Spatial frequencies are recovered by ssOS to approximately the same extent as by two-beam widefield SIM with an implementation of optical sectioning. The optical sectioning capability is furthermore exemplified by the filled *missing cone* of spatial frequencies along the axial frequency direction *k*_*z*_ displayed in Fig. 2e and f.

### Multiplexing with fluorescence anisotropy

The emitted light of fluorophores is often considered to be isotropic in terms of polarization. Consider a single fluorophore that acts as a dipole and randomly rotates in any direction on time scales shorter than the fluorescence lifetime. Due to this fast rotation, the dipole character does not affect the ensemble response of the fluorophore. Similarly, the excitation polarization has no effect if the fluorophore rotation is fast, i.e. happens on shorter time scales than the duration of an excitation pulse. In the case of short lifetimes and small rotational diffusion coefficients, however, polarized illumination preferentially excites fluorophore dipoles that are aligned and the emitted fluorescence retains a certain portion of polarization as well^10^. This is known as fluorescence anisotropy and the basis for fluorescence anisotropy imaging microscopy, FAIM. FAIM is conventionally used to measure rotational diffusion^10^, polymerization of proteins^11^ and intra-molecular changes^12^. Fluorescent proteins are known to have high anisotropy values *r* close to the theoretical maximum of *r* = 0.4 and are therefore well suited to realize the simultaneous projection of two structured illumination patterns encoded by orthogonal polarizations in living systems. The modulation contrast achievable with such a set-up can be calculated as follows. Creating and imaging two phase shifted SI patterns simultaneously via fluorescence anisotropy requires illumination of the sample *s* with a coarse stripe pattern *e*_*p*_ that features a period *p* = *f* × *k*_*c*_ under p-polarization and simultaneously with a out-of-phase stripe pattern of the same spatial frequency but in s-polarization. *k*_*c*_ is the cut-off frequency of the imaging objective. Note that depolarization effects by high numerical aperture *N*_*A*_ objectives are negligible as long as the excitation beams are chosen to be close to the optical axis in the objective back focal plane. The value of *f* should be smaller than 50% to achieve the best optical sectioning performance. Taking into account a limited modulation contrast *m* and a global phase offset *ϕ*_0_, the normalized excitation patterns are

9 $${e}_{p}(x)=\frac{1+m\,\cos (x\times p+{\varphi }_{0})}{1+m}$$

10 $${e}_{s}(x)=\frac{1+m\,\cos (x\times p+{\varphi }_{0}+\pi )}{1+m}.$$

For the reasons stated, it is justified to assume a high fluorescence anisotropy value *r* for fluorescent proteins. In the ideal case of *r* = 0.4, the ratio of the signal components measured in parallel and perpendicular directions to the excitation field is 3:1^10^. For the general case of arbitrary anisotropies, let us denote this ratio as *t*. The fluorescent emission is then split with a polarizing beam splitter to yield two images *i*_*p*_ and *i*_*s*_:

11 $${i}_{p}(x)=h(x)\otimes [t(s(x)\times {e}_{p}(x))+(s(x)\times {e}_{s}(x))]$$

12 $${i}_{s}(x)=h(x)\otimes [t(s(x)\times {e}_{s}(x))+(s(x)\times {e}_{p}(x))].$$

where *h* is the point spread function. Inserting the expressions for the excitation patterns *e*_*s*_ and *e*_*p*_ yields

13 $$\begin{array}{rcl}{i}_{p}(x) & = & h(x)\otimes [t(s(x)\times \frac{1+m\,\cos (x\times p+{\varphi }_{0})}{1+m})\\  &  & +\,(s(x)\times \frac{1+m\,\cos (x\times p+{\varphi }_{0}+\pi )}{1+m})]\end{array}$$

14 $$\begin{array}{rcl}{i}_{s}(x) & = & h(x)\otimes [t(s(x)\times \frac{1+m\,\cos (x\times p+{\varphi }_{0}+\pi )}{1+m})\\  &  & +\,(s(x)\times \frac{1+m\,\cos (x\times p+{\varphi }_{0})}{1+m})]\end{array}$$

and a combination of the two excitation pattern contributions results in a single harmonic excitation pattern for both recorded images:

15 $$\begin{array}{l}{i}_{p}(x)=h(x)\otimes [s(x)\times (\frac{1+t}{t(m+\mathrm{1)}}-\frac{m\mathrm{(1}-t)}{t(m+\mathrm{1)}}\,\cos (x\times p+{\varphi }_{0}))]\end{array}$$

16 $$\begin{array}{l}{i}_{s}(x)=h(x)\otimes [s(x)\times (\frac{1+t}{t(m+\mathrm{1)}}+\frac{m\mathrm{(1}-t)}{t(m+\mathrm{1)}}\,\cos (x\times p+{\varphi }_{0}))]\mathrm{.}\end{array}$$

The effective excitations ${e^{\prime} }_{s}$ and ${e^{\prime} }_{p}$ can be expressed as

17 $${e^{\prime} }_{p}(x)=\frac{1+t}{t(m+\mathrm{1)}}-\frac{m\mathrm{(1}-t)}{t(m+\mathrm{1)}}\,\cos (x\times p+{\varphi }_{0})$$

18 $${e^{\prime} }_{s}(x)=\frac{1+t}{t(m+\mathrm{1)}}+\frac{m\mathrm{(1}-t)}{t(m+\mathrm{1)}}\,\cos (x\times p+{\varphi }_{0})$$

and, assuming the best case of *t* = 3 and *m* = 1 can be written more concisely as

19 $${e^{\prime} }_{s/p}=\frac{2}{3}\pm \frac{1}{3}\,\cos (x\times p+{\varphi }_{0}\mathrm{).}$$

This translates into a modulation contrast of 50%, which is acceptable for conventional 2D SIM implementing optical sectioning^13^. The recorded raw image spectra using the excitation patterns stated in Equation 10 are then

20 $$\begin{array}{rcl}{I}_{s/p} & = & H(k)\times (S(k)\otimes {E}_{s/p}(k))\\  & = & \frac{1}{1+n}H(k)\times (S(k)\pm nS(k-p){e}^{-i{\varphi }_{0}}\mp nS(k+p){e}^{i{\varphi }_{0}}).\end{array}$$

**Figure 3:**
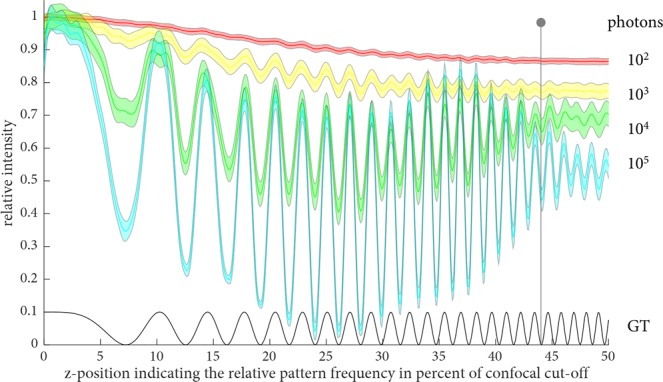
Analysis of the noise performance of single-shot OS. The ground truth pattern (GT), is scaled by 0.1 for better visibility. GT increases in axial spatial frequency from zero to 50% of the axial confocal spatial frequency cut-off. Single-shot OS reconstructions with various signal levels (10^5^ to 10^2^ photons in the brightest pixel) are displayed. The highest signal level is shown in cyan, and decreasing orders of magnitude are displayed in the green, yellow, and red curves. Shaded areas indicate the standard deviations of 500 repeats. The grey vertical line shows the theoretical axial cut-off.

Equation 20 is exactly the format required for ssOS as employed in the derivation starting with Equation 2 of the manuscript. Care must be taken when using high *N*_*A*_ objectives as well as with regard to the quality of the employed beam splitters as they can influence the overall observed anisotropy and the achievable extinction ratio^14^. Several correction factors can be calculated to counter these effects in FAIM imaging, but are of lesser interest in OS SIM, where the emphasis is on a clean separation of the two illumination patterns. Nevertheless, precise alignment is paramount to retain the best possible performance^15^. Furthermore, in logical consequence to the presented calculations on achievable modulation contrast with fluorescence anisotropy, the more general question arises of how ssOS performance is affected by low prevailing signal levels. Hence a further simulation was performed with varying signal levels using a Poisson noise model. This was investigated for the *in silico* sample presented in Fig. 2 using the same simulation parameters for image formation. In a noise-performance experiment, summarized in Fig. 3, four different signal levels (10^5^ to 10^2^ photons in the brightest pixel) were compared to a ground truth signal. The simulations show that an axial resolution up to the theoretical limit can be achieved when more than 10^4^ photons are detected. At light levels in the order of 10^2^ photons, noise prohibits the faithful recovery of axial spatial frequency information. The cross-over point is around 10^3^ photons, where only some axial spatial frequencies can be reconstructed.

### Practical considerations and experimental realization

**Figure 4:**
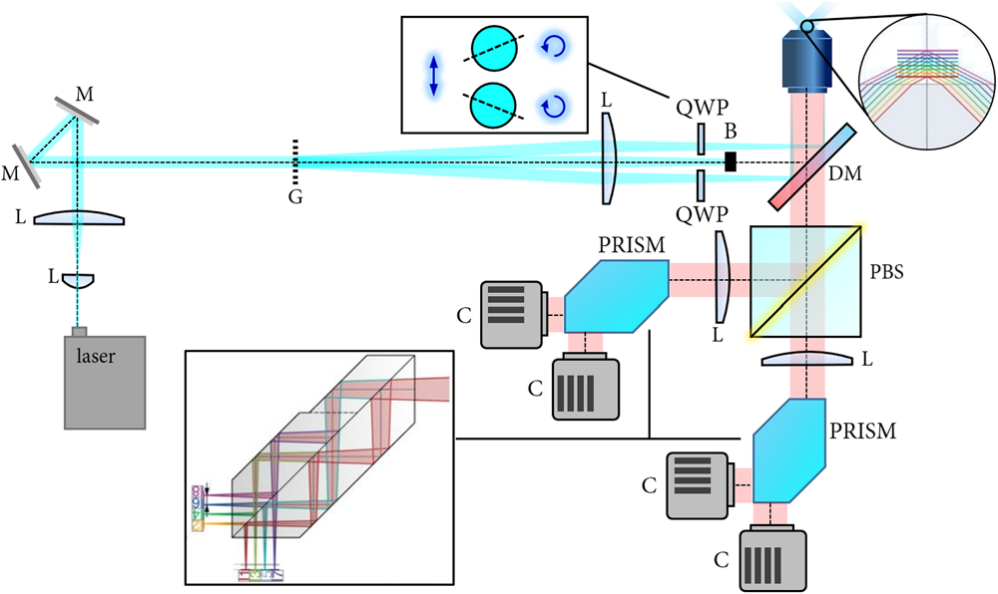
Example setup for volumetric imaging with ssOS. Details are provided in the text. The inlay depicting the effect of the multi-plane prisms (PRISMs) were adapted from^18^.

An optical setup for single shot volumetric imaging is depicted in Fig. 4. It can be broadly broken down into three main parts that comprise pattern generation, separation of polarization-coded sub-frames, and volumetric image capture.

#### Pattern generation

A vertically polarized beam from a laser is expanded by two two lenses (L) in a telescope configuration to cover the illumination volume of interest and relayed via two mirrors (M) onto a grating (G), which produces multiple diffraction beamlets. Another lens (L) captures the diffraction orders up to the first order and focuses them onto the back focal plane of the objective lens. The zeroth order beam is blocked with a beam block (B), and the first order beams pass through quarter wave plates (QWPs), which have their fast axis orthogonal to each other and at ±45° with respect to the polarization direction of the incident beams. Note that the two QWPs should be from the same batch to allow interference from low-coherence excitation sources over the full observation volume. Using this arrangement, the two beamlets become orthogonally polarized to one another and will hence not produce *intensity* interference fringes in the sample plane but an overall homogeneous illumination. As the beams are in a *σ*^+^–*σ*^−^ configuration^16^ (very similar to the lin-perp-lin configuration used in laser cooling applications^16^), they will, however, produce interference fringes in terms of their total electric field components, i.e. both interfering, but orthogonal, polarization directions produce intensity fringe patterns that are out-of-phase with each other. This is exactly the arrangement needed for ssOS.

#### Separation of sub-frames

The separation of the two patterns is achieved by a polarizing beam splitter (PBS) placed in the infinity path of the microscope after a dichroic mirror (DM) to split excitation and emission light. Alternative methods of raw-frame separation are thinkable and will be discussed later. Respective excitation and emission filters are not shown separately in the diagram. Note that the PBS needs to be aligned with the polarization of the excitation laser. The PBS is followed by two tube lenses, one in each arm, to produce an image of the sample on the cameras. To allow real-time volumetric imaging of multiple sections simultaneously, yet another optical component is necessary to collect multiple focal planes at an instance.

#### Volumetric imaging

Various implementations to enable multi-plane imaging have been described in the literature. The most prominent are multifocus gratings^17^, multi-plane prisms (PRISM)^18^ and multi-channel beam splitter arrangements^19^. In the proposed optical layout, two PRISM elements are used in conjunction with four cameras (C) as this arrangement permits the recording of eight planes under two polarizations simultaneously, while minimizing chromatic aberrations of the fluorescent emission.

## Experimental Results

**Figure 5:**
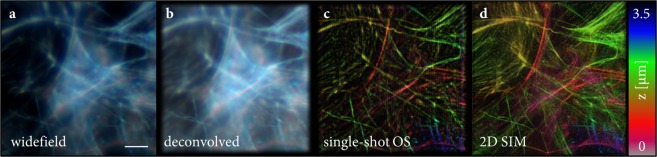
A z-stack of SIM raw data of actin-stained fibroblast cells was taken on the microscope described in^20^. (**a**) A widefield stack was generated by averaging all raw frames per slices and (**b**) RL deconvolution was employed. (**c**) A single-shot OS raw data stack was generated from the same data using only data from the *x*-modulated orientation and averaging two phase stepped images in order to produce -shifted raw frames in that direction. (**d**) Conventional 2D SIM with optical sectioning was reconstructed using the full data set and the ImageJ plugin FairSIM^13^. The scale bar is 5 m and color indicates focal planes.

The theoretical framework of the ssOS algorithm was validated by application to experimental data of fixed actin-stained fibroblasts, labeled with Alexa Fluor^©^ 488. To obtain raw data that can be expected from our proposed system, we used a custom-built SIM microscope^20^ in conventional 2-beam SIM mode to image the sample and used the acquired data set to *emulate* ssOS raw data. From the SIM raw data a conventional optically sectioned 2-beam SIM image was reconstructed using the ImageJ plug-in FairSIM^13^. The same raw data set was also averaged to yield a set of widefield images, which were deconvolved slice-wise using Richardson-Lucy deconvolution (code published elsewhere^15^). To produce a suitable data set for the proposed ssOS algorithm, the first phase of the first orientation in each slices was used as image *i*′ and the two images with phase-stepped patterns were averaged to yield the required raw image *i*″ phase-shifted by. Note that this keeps the same wave-vector but alters the modulation of the second image *i*″. As the modulation is calculated from the raw data, this is without influence on the actual reconstruction algorithm. Figure 5 displays the results of a stack produced from 15 slices with depth coded in color. Note that the pattern parameters were retrieved by the implemented ssOS algorithm from the structured illumination raw data only with pixel precision (rather than sub-pixel precision) in Fourier space. In conjunction with low contrast this might be the cause of the stripe artifacts visible in the ssOS image.

## Discussion and Conclusion

As deconvolution of widefield images can only guess axial spatial frequency information due to the missing cone, a method of optical sectioning microscopy was sought that can be implemented as a single-shot imaging technique akin to deconvolution microscopy. Using polarization imaging in conjunction with structured illumination, the framework for such a technique was derived and proof-of-concept imaging performed *in silico*. Experimental validation of the ssOS reconstruction algorithm was furthermore performed on fixed cells.

Using a range of simulations, it was found that an axial resolution extent up to the theoretical limit can be achieved when more than 10^4^ photons are detected. In the cell-imaging experiments the attention was put on the performance of ssOS in a challenging environment and in direct comparison to deconvolution microscopy and SIM. Here, pure deconvolution manages to emulate optical sectioning only to a limited extent (see e.g. the cells on the left-hand side of Fig. 5b), which can be attributed to a certain degree of axial spatial frequency support at higher lateral spatial frequencies via the high *N*_*A*_ of the employed objective. Using ssOS, on the other hand, a clear visual improvement similar to conventional 2-beam SIM with optical sectioning is seen. Note that we used a low modulation contrast of the illumination pattern, measured to be 0.1, to emulate the performance of polarization-coded fringes. Despite this obstacle, we were still able to effectively optically section the reconstructed volumes, which highlights the potential of the proposed technique.

A thorough analysis of an optical setup was also conducted, which allows the acquisition of whole volume data-stacks suitable for the proposed sectioning algorithm in a single shot. Note, that the simultaneous generation of different illumination patterns necessary for ssOS can theoretically be realized via a multitude of ways. Here, the use of orthogonal polarization was highlighted, as its implementation is straightforward and requires no active components. However, when labels with inherent directionality are to be used, e.g. intercalating dyes along a filament that would yield a non-isotropic fluorophore intensity response, methods other than polarization could be better suited to differentiate the illumination lattices within single acquisition frames. For example, differentiation could be achieved by intensity modulation of the illumination patterns during the acquisition time window and using phase sensitive detector arrays to discriminate the two patterns. If the intensity of the two patterns were modulated at frequencies *ω*_1_ and *ω*_2_, then use of two cameras demodulating at *ω*_1_ and *ω*_2_, would permit the discrimination of the two excitation lattices. As a thought experiment, let us assume that 1000 modulation cycles are required to achieve good pattern discrimination. For a 10 ms exposure time this would require a modulation frequency of 10^5^ Hz. Such technology is readily available in practice and used, for example, in frequency domain lifetime imaging, FLIM, where modulation frequencies in the 10 s of MHz are used routinely^21^. Concepts developed in the past for FLIM^22^ may have potential to be adapted for the current application, to achieve on-chip, real-time, demodulation of the lattices during image acquisition^22^. A similar approach is possible by temporally interleaving the two patterns and using a triggered phase-sensitive camera (e.g. pco.FLIM as used in^23^) to separate the two patterns. Each pixel in such a camera has two charge-collection taps that are filled independently and which can be opened and shut at sub-millisecond rates. During the exposure time of the camera, two patterns are projected onto the sample at the same frequency *ω* but with a 180° phase-shift. Summation of signal from pattern 1 in the first tap and pattern 2 in the second tap via phase-sensitive switching enables the separation of the patterns. Multi-color labeling to realize a simultaneous pattern projection and detection is also a possibility. In such an implementation, the structure under investigation has to be co-labeled with two different fluorophores, which permits excitation and emission in separate spectral bands. Although two-color labeling is simple in principle^24^, it is difficult in practice as the required spatial frequencies of the patterns need to be equal in direction and magnitude. Furthermore, the detection of different colors inevitably results in different optical transfer function supports, which are not covered explicitly by the proposed sectioning algorithm.

As the presented optical sectioning technique is based on a purely linear process, its use in quantitative imaging is straightforward. Furthermore, as only two raw frames are required, it can be realized as a single-shot imaging method via orthogonally polarized excitation patterns in conjunction with a polarization-sensitive detection. The striking benefit of ssOS in comparison to other high frame-rate single-shot optical sectioning techniques like spinning-disk confocal microscopy is the possibility of multi-plane acquisitions. As the illumination patterns required for ssOS are invariant along the optical axis, it is conceivable to record entire sectioned volumes through multi-focus microscopy^17,18^. Given sensitive and fast enough cameras and sufficiently high modulation contrast, this approach might rival the speed and gentleness of cellular volumetric imaging with selective plane illumination microscopy (SPIM). Benefits of volumetric ssOS are numerous but particularly promising in multi-particle tracking studies. As the efficiency of single point emitter photon collections is dependent on the solid angle of the objective rather than the *N*_*A*_, much higher photon collection efficiencies are possible because higher *N*_*A*_ objectives than for example in SPIM can be realized. Furthermore, as whole sectioned volumes could be captured at an instance, reconstruction artifacts due to motion blur between frames would be minimized.

## Materials and Methods

### Simulation parameters

In the simulation presented in Figs 2 and 3 the spatial frequency increased from zero at the top to the confocal spatial frequency cut-off at the bottom using the cut-off frequency formula given by Amos *et al*.^25^:

21 $${\rm{\Delta }}z=\frac{0.64{\lambda }_{em}}{n\sqrt{{n}^{2}-{N}_{A}^{2}}}.$$

The simulation used an emission wavelength *λ*_*em*_ of 520 nm and a 1.49*N*_*A*_ in conjunction with oil immersion of refractive index *n* = 1.518. The modulation contrast of the structured illumination was 0.5 and its spatial frequency at 30% of the lateral excitation cut-off. An illumination wavelength of 488 nm was set.

### Structured illumination microscope

To obtain experimental data, we used an existing custom-built structured illumination microscope. For pattern generation of ssOS and 2-beam SIM we used a spatial light modulator (SLM)^20^. For excitation of Alexa Fluor© 488 fluorescence, we used a 488 nm diode laser (IBEAM-SMART-488, Toptica), and imaged emission via a quad-band dichroic mirror (ZT405/488/561/640rpc, Chroma) and a band pass filter (FF01-525/30-25, Semrock) onto an sCMOS camera (ORCA Flash 4.0, Hamamatsu). A water immersion objective lens (UPLSAPO60XW, 60×/1.2*N*_*A*_, Olympus) was used with an additional 1.6× magnification to give a total magnification of 96×. This resulted in an imaging pixel size of 65 nm and total field of view of 6 5× 65 *μm*^2^. 2-beam SIM gratings displayed on the SLM resulted in a line spacing of 381 nm at the sample, corresponding to SIM beamlet positions at 50% of the radius of the objective back aperture. An exposure time of 100 ms was used per raw SIM frame. To acquire 3D stacks we used an automated *xy* stage equipped with a *z* axis piezo (PZ-2150, Applied Scientific Instrumentation) to move the sample position relative to the objective lens. We took 15 axial planes and for each plane of the 3D stack, nine raw images were acquired with three phase steps per pattern orientation (0deg, 60deg and 120deg), and the axial step size was set to 250 nm. For image reconstruction of optically sectioned 2-beam SIM images FairSIM^13^ was used. To produce the data set for ssOS, the first phase of the first orientation in each slices was used as image *i*_*p*_ and the two images with 3*π*/2 phase-stepped patterns were averaged to yield the required *π* phase-shifted raw image *i*_*s*_.

### Fibroblast sample

For cell imaging, fixed actin-stained fibroblasts labeled with Alexa Fluor^©^ 488 (Thermofisher Scientific) via indirect immunohistochemistry were used.

## Data Availability

Data and software are available on request.
